# The effects of chiropractic spinal manipulation on central processing of tonic pain - a pilot study using standardized low-resolution brain electromagnetic tomography (sLORETA)

**DOI:** 10.1038/s41598-019-42984-3

**Published:** 2019-05-06

**Authors:** Muhammad Samran Navid, Dina Lelic, Imran Khan Niazi, Kelly Holt, Esben Bolvig Mark, Asbjørn Mohr Drewes, Heidi Haavik

**Affiliations:** 1 Mech-Sense, Department of Gastroenterology and Hepatology, Aalborg University Hospital, Aalborg, Denmark; 2 Department of Clinical Medicine, Aalborg University, Aalborg, Denmark; 3 Centre for Chiropractic Research, New Zealand College of Chiropractic, Auckland, New Zealand; 4 Faculty of Health & Environmental Sciences, Health & Rehabilitation Research Institute, AUT University, Auckland, New Zealand; 5 Centre for Sensory-Motor Interactions (SMI), Department of Health Science and Technology, Aalborg University, Aalborg, Denmark

**Keywords:** Sensorimotor processing, Sensory processing, Health care

## Abstract

The objectives of the study were to investigate changes in pain perception and neural activity during tonic pain due to altered sensory input from the spine following chiropractic spinal adjustments. Fifteen participants with subclinical pain (recurrent spinal dysfunction such as mild pain, ache or stiffness but with no pain on the day of the experiment) participated in this randomized cross-over study involving a chiropractic spinal adjustment and a sham session, separated by 4.0 ± 4.2 days. Before and after each intervention, 61-channel electroencephalography (EEG) was recorded at rest and during 80 seconds of tonic pain evoked by the cold-pressor test (left hand immersed in 2 °C water). Participants rated the pain and unpleasantness to the cold-pressor test on two separate numerical rating scales. To study brain sources, sLORETA was performed on four EEG frequency bands: delta (1–4 Hz), theta (4–8 Hz), alpha (8–12 Hz) and beta (12–32 Hz). The pain scores decreased by 9% after the sham intervention (*p* < 0.05), whereas the unpleasantness scores decreased by 7% after both interventions (*p* < 0.05). sLORETA showed decreased brain activity following tonic pain in all frequency bands after the sham intervention, whereas no change in activity was seen after the chiropractic spinal adjustment session. This study showed habituation to pain following the sham intervention, with no habituation occurring following the chiropractic intervention. This suggests that the chiropractic spinal adjustments may alter central processing of pain and unpleasantness.

## Introduction

Changes in the way the human brain processes pain, as well as the capacity to modulate the pain experience, underlies the pathogenesis of most chronic pain conditions^1^. Typically, the perception of pain is induced by a potential damaging stimulus in the periphery, which activates peripheral nerves. The pain signal is transmitted to the spinal cord and further to deep centers within the brain. However, the pain system is not hard-wired, but rather a complex dynamic process with advanced modulatory properties, such as activation of descending pathways that can modulate pain perception, and that is also responsible for the recurrence and chronicity of the pain experience^2–4^. During the last two decades of research, there has been a growing interest to understand the underlying mechanisms of the descending inhibition of pain. Pain modulating processes in humans are present at multiple levels of the human nervous system; at the spinal level in the dorsal horn^5^, in the brainstem, including neurons in the rostral ventromedial medulla and periaqueductal grey (RVM-PAG)^6^, and at the cortical level^7^. Cognitive and affective processes within the limbic system are also known to influence the RVM-PAG system, and are also involved in the top-down cortico-limbic-brainstem inhibition^8,9^. In the literature, these kinds of central nervous system modulations of the pain experience are collectively known as conditioned pain modulation (CPM).

Chiropractic is one option that is utilized clinically to help people who suffer with musculoskeletal pain, both acute and chronic. Multiple clinical and basic science studies have shown that chiropractic care can reduce pain^10–12^, however the mechanisms involved are not well understood^13–16^. Chiropractic care is a holistic approach to health with a particular focus on the relationship between the spine and nervous system^17^. Traditionally, the main focus of chiropractic care has been the location, analysis and correction of vertebral subluxations^17^. Vertebral subluxations have been defined as ‘a self- perpetuating, central segmental motor control problem that involves a joint, such as a vertebral motion segment, that is not moving appropriately, resulting in ongoing maladaptive neural plastic changes that interfere with the central nervous system’s ability to self-regulate, self-organize, adapt, repair and heal’^18^. Chiropractors use a combination of indicators of spinal dysfunction to identify and characterize vertebral subluxations^19^. They then use a variety of manual techniques, the most common being specific high-velocity, low amplitude adjustments, to correct the vertebral subluxation^20,21^. This is sometimes referred to as spinal manipulation in the research literature, however, spinal manipulation is also used in the literature for other forms of non-specific thrusts delivered to spinal regions as opposed to specific dysfunctional vertebrae^22^. Research has shown that chiropractic spinal adjustments alter the afferent input from the spine which leads to changes in central nervous system (CNS) function^23^. It has been postulated that spinal adjustments stimulates the dysfunctional paraspinal tissues, which modulates central processing and potentially sensitization, and therefore alters the perception of pain cortically, in a ‘top-down’ manner or both^13,23–25^. The majority of the studies which have shown neuroplastic effects following a chiropractic intervention have focused on changes in somatosensory processing, motor control and functional performance^26–29^. These neural plastic changes have been assessed with both spinal and cortical measures^30,31^, however, knowledge about the effects of spinal adjustments on pain and neural markers for altered CNS function due to this alteration in sensory input from the spine is still lacking^14,32^.

As it is becoming clear that chiropractors impact brain function consistently, it is very likely that chiropractic care influences not only the biomechanical movement patterns of the spine and improves proprioceptive processing of the spine^33,34^ but also directly impacts the so called ‘pain matrix’ in the brain and thus has a CPM effect on a person’s perception of pain. Chiropractic care may alter the way a person ‘feels’ pain and may therefore help chronic musculoskeletal pain sufferers by improving the top-down cortico-limbic-brainstem inhibition of pain. Alternatively, chiropractic spinal adjustments may also alter pain perception via the neuroendocrine system, or segmental inhibition at the spinal cord level, or even motivational-affective modulation^10,13,23,24,32^. Further research is therefore required to elucidate the basic neurophysiological mechanisms of spinal adjustments and their effect on pain perception^13,14,32^.

Tonic pain models can be used in experiments to study pain, as experimental tonic pain produces a similar sensory experience to that of a clinical setting^35–37^. The cold-pressor (CP) test has been shown to be a robust stimulus to induce tonic pain^38^ and mimics clinical pain due to its high level of unpleasantness^39^. Using the CP test together with electroencephalography (EEG), Gram *et al*. showed that EEG spectral indices change in parallel with pain intensity^38^, further validating this method to assess central processing of tonic pain. Shao *et al*., using EEG and standardized low-resolution brain electromagnetic tomography (sLORETA), showed that tonic cold pain induced significant changes of source power across different frequency bands in many brain regions, including the prefrontal, primary and secondary somatosensory (S1 and S2), insular and cingulate cortices^40^. These same regions have been consistently shown to be activated or deactivated during pain perception by various neuroimaging studies using functional magnetic resonance imaging (fMRI), magnetoencephalography (MEG) and positron emission tomography (PET) (see e.g.^7,41–43^). Specifically, Shao *et al*. found that the oscillatory activities that significantly correlated with subjective pain ratings were found in the prefrontal and cingulate regions^40^. The authors recommended the testing of pain treatment methods, especially to explore whether they could alter the neural oscillations in the particular frequency bands in the specific brain regions they had identified in their study. These specific brain regions and frequency bands identified during the CP test were the 4–8 Hz (theta) oscillatory activity in the prefrontal cortex, the 8–12 Hz (alpha) oscillatory activity in the anterior cingulate cortex (ACC) and the 12–18 Hz (beta) oscillatory activity in the posterior cingulate cortex (PCC).

In the present study, sLORETA was therefore used for source localization of EEG recorded pre and post chiropractic spinal adjustments and a sham intervention. The sLORETA is a linear inverse algorithm that estimates the 3D distribution of the cortical generators of the EEG and provides the lowest localization error compared to several other linear inverse algorithms^44^. The sLORETA was done on four frequency bands: (i) delta (1–4 Hz), (ii) theta (4–8 Hz), (iii) alpha (8–12 Hz), and (iv) beta (12–32 Hz). These frequency bands were chosen to cover the frequency changes noted by Shao *et al*.^40^ and because they have been the most commonly reported with LORETA analysis in frequency domain^45–47^.

The hypothesis of the current cross-over study was that a single session of chiropractic spinal adjustments would alter the neural response to tonic pain, in comparison to a sham spinal adjustment session. Therefore, the aims of this pilot study were to:

1. Assess whether there are differences in subjective pain perception and unpleasantness levels during the CP test following sham and chiropractic interventions, and
2. Compare the brain activity (sLORETA solution) underlying different frequency bands of the CP-EEG data before and after each intervention.

## Methods

The study was conducted according to the Declaration of Helsinki. The North Denmark Region Committee on Health Research Ethics approved the study (N-20150033). The study was one-way blinded; therefore, the participants did not know which type of intervention they received. This study was registered retrospectively on 24^th^ August 2018 with the Australian New Zealand Clinical Trial Registry (trial registration number ACTRN12618001420235). The study design is given in Supplementary Figure S1.

### Subjects

Fifteen subclinical pain subjects (10 males, (mean ± SD) age = 32.1 ± 7.2 years, BMI = 24.02 ± 5.94 kg/m^2^) participated in the study. Subclinical pain refers to recurrent spinal dysfunction such as mild pain, ache or stiffness for which treatment is not yet sought, and most importantly no pain on the day of experimental assessment, to avoid the confounding effects of altered resting pain levels. All subjects gave their written informed consent to participate in the study.

Before entering the study, the subjects were introduced to the lab environment and were assessed by a chiropractor with 15 years of experience to assure that the subjects pass the eligibility criteria to participate in the study. Subjects were included if they were aged between 18 and 50 years, had a history of recurring spinal dysfunction such as mild pain, ache or stiffness without a history of known trauma. Subjects were ineligible to participate if they exhibited actual pain on the day(s) of the experiment, had no evidence of spinal dysfunction, had absolute contraindications to chiropractic spinal adjustments, had experienced previous significant adverse reactions to chiropractic care or spinal manipulation, or if they had sought treatment for their pain symptoms. The subjects were also required to be fluent in the understanding of written and spoken English to participate in the study.

### Experimental protocol

**Figure 1:**
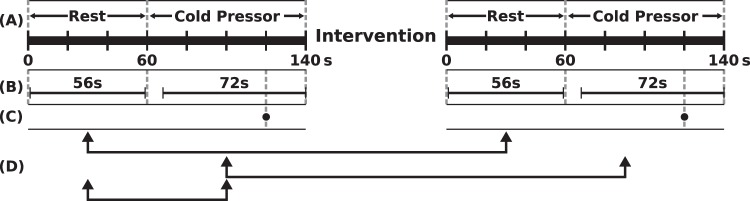
Methodology overview. (**A**) Shows the order and duration of the resting state and cold- pressor test during which EEG was also recorded. (**B**) Shows the length and position of EEG used for analysis after it was truncated. The circle in (**C**) shows the time point when the participant rated the pain and unpleasantness to cold-pressor. (**D**) Shows the pairs used for comparisons in sLORETA. The intervention lasted approximately 10 minutes. Abbreviations: sLORETA = standardized low-resolution brain electromagnetic tomography.

The subjects participated in two experimental sessions; sham and chiropractic, separated by 4.0 ± 4.2 days at Aalborg University Hospital. Each session consisted of a 1-minute resting state EEG recording, followed by a CP test during which EEG was also recorded. The resting EEG was recorded in order to observe the central processing of tonic pain itself (i.e. difference between pre-intervention CP-EEG and pre-intervention resting EEG) and this would subsequently aid in interpreting the changes in the central processing of tonic pain following the interventions. Figure 1 shows the overview of the experiment.

### Interventions

The chiropractic spinal adjustment intervention and sham intervention were similar to those used in previous studies that have investigated the neurophysiological effects of chiropractic spinal adjustments^23,30,31^. The two researchers who carried out the interventions made a concerted effort to maintain the same levels of language and professional dealing with the participants. We advertised about the recruitment on a Facebook page. All the participants (upon questioning) were completely naïve to chiropractic care, so had no previous experience with a chiropractor, and therefore did not know what to expect. Participants were blinded to nature of intervention (sham or adjustment intervention). At the end of the second session, before telling them which session a real chiropractic intervention was, and which one was a sham intervention, the participants were asked whether they thought one session could have been a sham. Out of the 15 subjects, three subjects said that one session felt like a sham. Out of these three, two were correct and one was wrong, as this third person thought the sham was real and the real session was a sham.

### Chiropractic spinal adjustment intervention

The chiropractic spinal adjustments carried out were high-velocity, low-amplitude thrusts to the spine or pelvic joints, which is a standard adjustment technique used by chiropractors and is also referred to as spinal manipulation. The sites chosen for the spinal adjustments were based on the clinical indicators of spinal and pelvic joint dysfunction^19^, which were: tenderness to palpation of the relevant joints; manual palpation for restricted intersegmental range of motion; palpable asymmetric intervertebral muscle tension, and any abnormal or blocked joint play and end-feel of the joints. These indicators have been shown to be reliable when used in combination to identify level of subluxation in the spine^48^. Multiple levels of the spine were adjusted in each participant by a registered chiropractor during the chiropractic adjustment session.

### Sham intervention

The sham intervention acted as a physiological control. During the sham intervention, the chiropractor simulated a spinal adjustment session, which included passive and active movements of the participants head, spine, and body, similar to what is done during the chiropractic adjustment session. Care was taken during the sham session to not take any joints to their end range of motion or to cause a cavitation in the spine. This was done to limit the afferent barrage from large diameter afferents in the paraspinal muscles to the central nervous system, while controlling for the body movements, touch and vestibular input associated with setting up to provide chiropractic adjustments.

### Cold-pressor test

The CP test was performed using a circulating water bath (Grant, Fischer Scientific, Slangerup, Denmark). The water was cooled to 2 °C and the subjects immersed their left hand in the water up to the wrist for 80 seconds (Fig. 1A).

### Pain and unpleasantness scores

The participants rated their pain and unpleasantness on two separate numerical rating scales after their hand had been in the water for 60 seconds (Fig. 1C). The two scales ranged from 0 (no pain/unpleasantness) to 10 (maximum pain/unpleasantness), and the experimenter noted down the scores.

### EEG

EEG was recorded at a sampling rate of 1000 Hz in a dimly lit room using a 61-channel cap (MEQNordic A/S, Jyllinge, Denmark) and Synamp system (Neuroscan Compumedics, El Paso, TX, USA). The reference electrode was just above AFz. During the recordings, the participants lay in a supine position and were instructed to relax, keep their focus on a point and reduce eye blinking.

The EEG was preprocessed offline. The following preprocessing steps were performed using Neuroscan 4.3.1 (Neuroscan, El Paso, TX, USA): (1) noisy channels were interpolated using their neighboring channels; (2) afterward, a 50 Hz notch filter was applied, (3) followed by a band-pass filter of 1 to 70 Hz. For the remaining preprocessing steps, MATLAB 2015b (The MathWorks, Inc., Natick, MA, USA.) was used: (4) EEG was truncated for further analysis: the resting EEG from 2 to 58 s was taken, and for the CP part, 72 s of EEG were taken, starting at 8 s from the onset of the stimulus (Fig. 1B). The first 8 seconds of CP-EEG data were removed to avoid artifacts including muscle contractions caused by the immediate unpleasantness after immersing the hand into the cold water^38^; (5) to reduce the computational load in the sLORETA matrix calculations, the filtered EEG was downsampled by a factor of 4; and finally, (6) for obtaining smooth power spectral density, the EEG was divided into epochs with length of 8 s to facilitate the averaging procedure in sLORETA.

### sLORETA

The underlying sources of the EEG were estimated using the sLORETA software package, version 20151222^44^ (available at http://www.uzh.ch/keyinst/loreta). The sLORETA was done in the frequency domain to localize neural oscillators on the average referenced EEG. The EEG was average referenced in the sLORETA software. Cross-spectral matrices for each subject were computed in sLORETA software for four frequency bands: (i) delta (1–4 Hz), (ii) theta (4–8 Hz), (iii) alpha (8–12 Hz), and (iv) beta (12–32 Hz). The cross-spectral matrices for each participant were then averaged as the input for sLORETA source analysis. The sLORETA software was used to estimate the statistical differences in brain activity (in the four EEG frequency bands) between:

1. The baselines of both experimental sessions for (i) resting state EEG and (ii) CP-EEG to make sure there were no unexpected differences,
2. The baseline CP-EEG and baseline resting state EEG to observe how the brain processes tonic pain (Fig. 1D),
3. The post-intervention and baseline EEG for (i) resting state and (ii) CP to find the effect of each intervention on neural activity (Fig. 1D).

The cortical gray matter was divided into 6239 voxels with a resolution of 5 mm^3^. The head model and electrode coordinates according to Montreal Neurological Institute average MRI brain map (MNI-152)^49^ were used.

### Spectral analysis

The EEG power spectral analysis was performed using FieldTrip toolbox^50^ to assist the sLORETA results. To estimate the differences in the power spectrum of the four EEG frequency bands between the conditions mentioned in the section above, the power spectra between 1 and 32 Hz of the EEG were calculated using Fourier basis with an Hanning window of 1 s, which was followed by computation of the average power of each frequency band.

### Statistics

The data are presented as mean ± SD unless otherwise indicated. The statistical significance threshold was *p* < 0.05.

Two-way repeated measures analysis of variance (ANOVA) was performed to identify the changes in the pain and unpleasantness scores with time (before and after) and intervention (sham and chiropractic) as the two factors. If the overall significance in the ANOVA test was found, all pairwise multiple comparisons procedures (Student-Newman-Keuls Method) were performed in order to assess where the differences were. The software package SigmaStat version 3.0 (SPSS Inc. Chicago, IL, USA) was used for the above statistical analysis.

The statistical analysis for source localization was done using the sLORETA software’s built-in statistics tool using statistical non-parametric mapping^51^ which adjusted for multiple comparisons by utilizing Fisher’s random permutation test with 5000 randomizations. To compare current sources in different frequency bands, paired two-tailed Student’s *t*-test was used to compare the baselines of chiropractic and sham sessions; the baseline CP and baseline resting state; and the post-sessions and baselines.

Non-parametric cluster-based permutation test^52^ was used to identify the differences in the EEG power spectrum between the baselines of chiropractic and sham sessions; the baseline CP and baseline resting state; and the post-sessions and baselines. The clusters were defined as two or more continuous channel-power pairs each with *p* < 0.05 from the paired two-tailed *t*-test with respect to the conditions. The *t*-values within each cluster were added to get the cluster-level statistics and the maximum of cluster-level statistics was used as the test statistic. A cluster was considered significant if its Monte Carlo probability for each tail exceeded the threshold of 0.025 compared to the reference distribution approximated by Monte Carlo method with 5000 permutations.

## Results

All fifteen of the enrolled subjects successfully completed the experiment and data from all subjects were used for the analysis.

### Pain and unpleasantness scores

The pain and unpleasantness scores of the CP test are summarized in Table 1. The time effects on the pain scores (*F*_*1,14*_ = 6.7, *p* < 0.05) and unpleasantness scores (*F*_*1,14*_ = 9.6, *p* < 0.05) were significant. The posthoc test revealed that the pain scores decreased after the sham intervention (*p* < 0.05), whereas the unpleasantness scores decreased after both interventions (both *p* < 0.05). There were no significant interactive effects present between the time and intervention.

**Table 1:** Pain and unpleasant scores.

Scores	Sham Intervention		Spinal Adjustment Intervention	
	Before	After	Before	After
Pain	8.33 ± 1.54	7.53 ± 1.73^*^	8.27 ± 1.39	7.77 ± 1.88
Unpleasantness	8.73 ± 1.62	8.07 ± 2.19^*^	8.53 ± 2.07	7.90 ± 2.45^*^

^*^Significant differences (*p* < 0.05).

### Effects on source location

The sLORETA analysis showed no differences in both the resting state EEG baselines and the CP-EEG baselines in all frequency bands (all *p* > 0.05).

**Figure 2:**
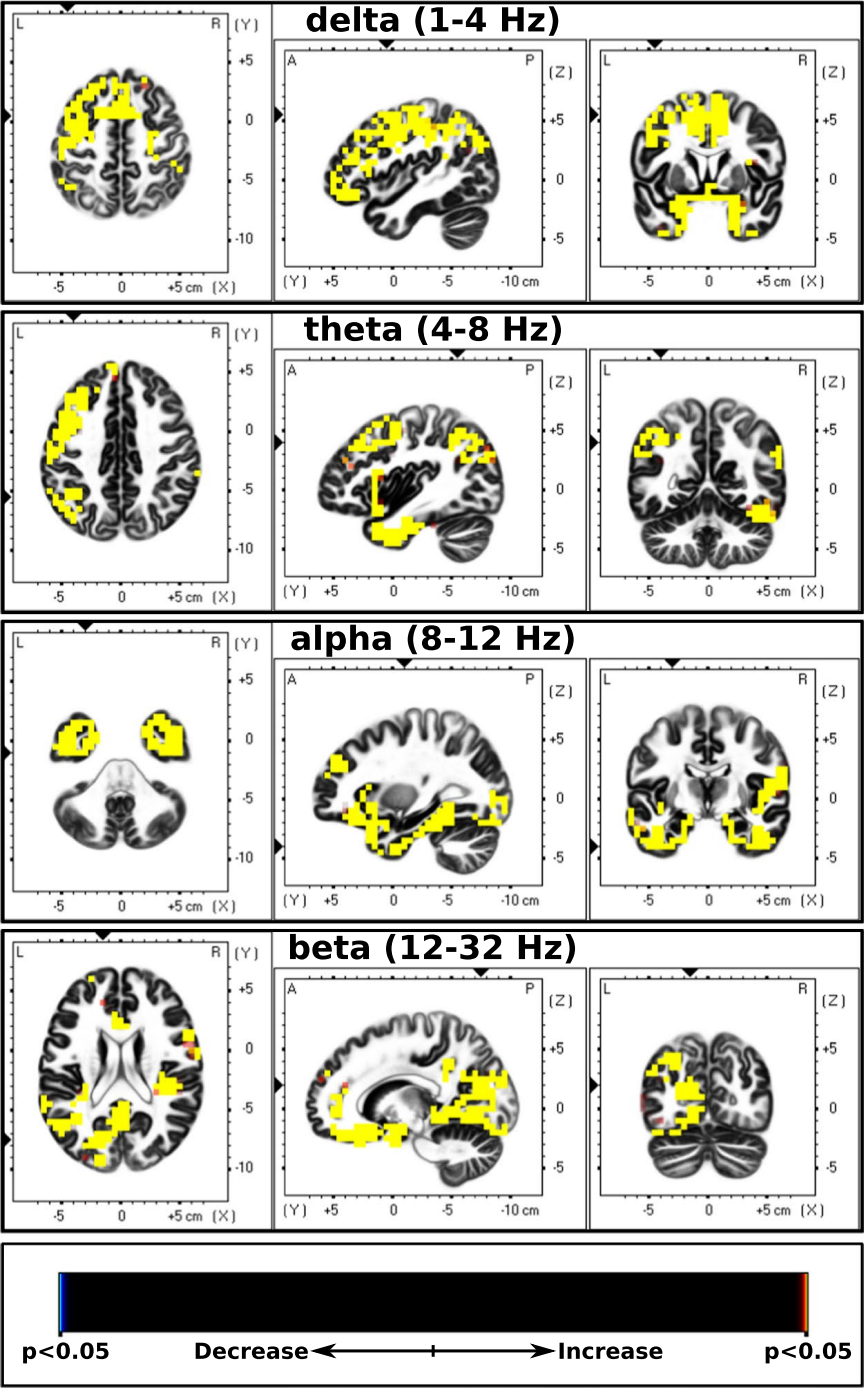
sLORETA CP vs Rest. Slice views of source locations with the changes in activity (t-values) between baseline cold-pressor and resting EEG for each frequency band. A significant increase (p < 0.05) in activity (yellow colored) in all frequency bands can be seen during the pain condition. Abbreviations: CP = cold-pressor; sLORETA = standardized low-resolution brain electromagnetic tomography.

The comparison between baseline CP-EEG and baseline resting state EEG showed a widespread increase in cortical activity during pain compared to resting state in all frequency bands. The results from the sLORETA analysis are summarized in Table 2 and Fig. 2.

**Table 2:** sLORETA localized EEG cortical sources with significant differences between the baseline cold-pressor and baseline resting state.

Brain structure (Brodmann Area)	Delta (1–4 Hz)	Theta (4–8 Hz)	Alpha (8–12 Hz)	Beta (12–32 Hz)
Angular Gyrus (39)	24	19	—	5
Anterior Cingulate (24, 25, 32, 33)	114	—	61	57
Cingulate Gyrus (23, 24, 31, 32)	192	—	—	38
Cuneus (17, 18, 19, 23, 30)	161	—	—	49
Extra-Nuclear (13)	—	—	10	—
Fusiform Gyrus (18, 19, 20, 21, 32, 37)	119	70	148	87
Inferior Frontal Gyrus (9, 10, 11, 13, 44, 45, 46, 47)	185	25	295	83
Inferior Occipital Gyrus (18, 19)	18	18	28	5
Inferior Parietal Gyrus (40)	—	—	—	25
Inferior Parietal Lobule (39, 40)	183	54	15	—
Inferior Temporal Gyrus (20, 37)	42	36	93	19
Insula (13)	68	22	64	35
Lingual Gyrus (17, 18, 19)	143	—	121	70
Medial Frontal Gyrus (6, 8, 9, 10, 11, 25, 32, 46)	97	—	92	39
Middle Frontal Gyrus (6, 8, 9, 10, 11, 46, 47)	276	95	121	13
Middle Occipital Gyrus (18, 19, 37)	68	51	65	—
Middle Temporal Gyrus (12, 21, 22, 37, 38, 39)	103	84	175	44
Orbital Gyrus (11, 47)	23	—	30	12
Parahippocampal Gyrus (19, 27, 28, 30, 34, 35, 36, 37)	172	56	101	139
Postcentral Gyrus (1, 2, 3, 40, 43)	116	32	6	—
Posterior Cingulate (23, 29, 30, 31)	85	—	13	60
Precentral Gyrus (4, 6, 9, 43, 44)	115	56	48	—
Precuneus (7, 9, 31)	55	—	—	69
Rectal Gyrus (11)	39	—	44	36
Subcallosal Gyrus (25, 34)	18	—	18	17
Sub-Gyral (6, 20, 21)	6	—	17	—
Superior Frontal Gyrus (6, 8, 9, 10, 11)	171	10	—	10
Superior Occiptial Gyrus (17, 19)	7	6	—	—
Superior Parietal Lobe (7)	—	6	—	—
Superior Parietal Lobule (7)	8	—	—	23
Superior Temporal Gyrus (19, 22, 38, 39, 41, 42)	146	78	229	86
Supramarginal Gyrus (40)	45	24	21	—
Transverse Temporal Gyrus (41, 42)	10	—	5	5
Uncus (20, 28, 34, 36, 38)	64	19	64	57

The number of voxels with significant power changes (*p* < 0.05) is listed.

Neither of the two interventions changed the resting state EEG (all *p* > 0.05).

**Figure 3:**
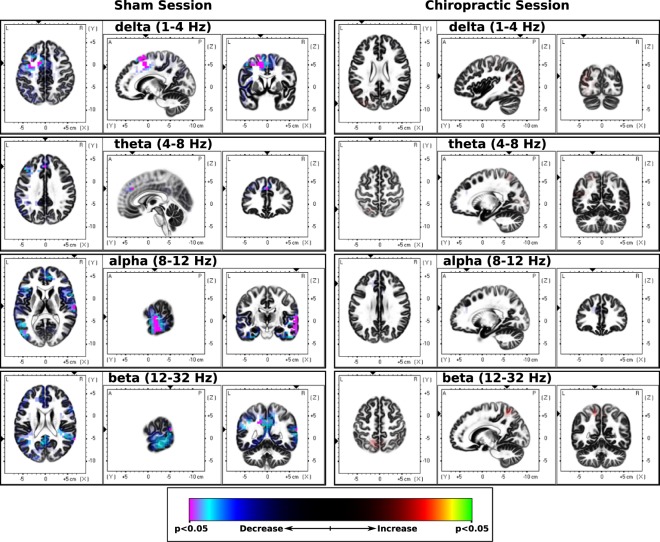
sLORETA CP(Treatment) vs CP (Baseline). Slice views of source locations with the changes in activity (t-values) during the cold-pressor test for each frequency band after sham and spinal adjustment sessions compared to respective baseline activity. The decrease in activity in all frequency bands can be seen after the sham session with a significant decrease (p < 0.05) in activity (magenta colored) in delta and alpha bands; and marginally significant decreases (p = 0.05) in the theta and beta bands. Abbreviations: CP = cold-pressor; sLORETA = standardized low-resolution brain electromagnetic tomography.

The brain activity underlying the CP test decreased following the sham intervention (Fig. 3 and Table 3) but showed no differences following the spinal manipulation. Following the sham session, the most significant decreases (*p* < 0.05) in activity due to CP were seen in the delta (Brodmann area 32, cingulate gyrus, limbic lobe) and alpha (Brodmann area 42, transverse temporal gyrus, temporal lobe) bands, whereas marginally significant decrease (*p* = 0.05) were seen in the theta (Brodmann area 9, medial frontal gyrus, frontal lobe) and beta (Brodmann area 22, superior temporal gyrus, temporal lobe) bands. The complete list of brain regions showing changes in activity can be seen in Table 3.

**Table 3:** sLORETA localized EEG cortical sources during the cold-pressor test with significant differences after sham session compared to baseline activity.

Brain structure (Brodmann Area)	Delta (1–4 Hz)	Theta (4–8 Hz)	Alpha (8–12 Hz)	Beta (12–32 Hz)
Cingulate Gyrus (24, 32)	5	—	—	—
Fusiform Gyrus (42)	—	—	1	—
Inferior Parietal Lobule (20)	—	—	1	—
Inferior Temporal Gyrus (20, 21, 22, 40)	—	—	4	—
Medial Frontal Gyrus (6, 9, 32)	4	1	—	—
Middle Frontal Gyrus (6)	7	—	—	—
Middle Temporal Gyrus (20, 21, 22, 40, 42)	—	—	9	—
Precuneus (31)	—	—	—	1
Sub-Gyral (6, 31)	1	—	—	1
Superior Frontal Gyrus (6)	10	—	—	—
Superior Temporal Gyrus (20, 22, 40)	—	—	7	1
Supramarginal Gyrus (21, 22)	—	—	6	—
Transverse Temporal Gyrus (21)	—	—	1	—

The number of voxels with significant power changes (p < 0.05) is listed for delta and alpha bands; and with marginal significant changes (*p* = 0.05) for the theta and beta bands.

### Effects on power spectrum

**Figure 4:**
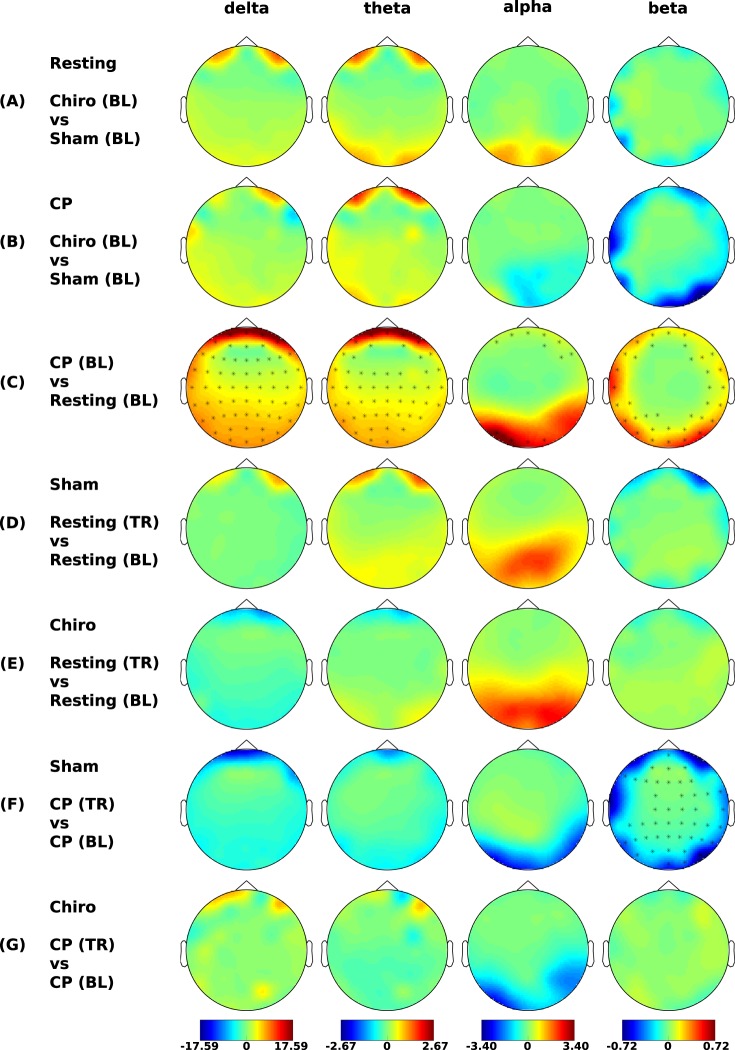
Spectral analysis. Differences in power (μV^2^/Hz) of the four frequency bands. No differences in (**A**) resting state and (**B**) CP-EEG baselines. (**C**) A significant increase (p < 0.05) in power (asterisks) in all frequency bands can be seen during the pain condition. No effect of both sham (**D**) and the chiropractic (**E**) interventions on the resting state. (**F**) The decrease in power of CP-EEG in all frequency bands can be seen after the sham session with a significant decrease (p < 0.05) in beta band. (**G**) There was slight increase in the overall power of CP-EEG in all frequency bands except alpha band after the chiropractic session. Abbreviations: CP = cold-pressor; BL = baseline; TR = treatment; Chiro = chiropractic.

Similar to the sLORETA results, the spectral analysis showed no differences in both the resting state EEG baselines (Fig. 4A) and the CP-EEG baselines (Fig. 4B) in all frequency bands (all *p* > 0.05).

The comparison between baseline CP-EEG and baseline resting state EEG showed a widespread increase in EEG power during pain as compared to resting state in all frequency bands (Fig. 4C).

Neither of the two interventions changed the resting state EEG power (Fig. 4D,E) (all *p* > 0.05).

The EEG power underlying the CP test decreased following the sham intervention (Fig. 4F), with significant decrease in the beta band. Although non-significant for the delta, theta, alpha band, the trend of decreased brain activity was similar to the sLORETA results. The EEG power underlying the CP test showed a slight (non-significant) increase following the spinal manipulation (Fig. 4G) in all frequency bands except the alpha band.

## Discussion

In this study, we investigated the effects of altering sensory input from dysfunctional areas of the spine (by spinal adjustments) on pain perception and the neural activity (in terms of source localization of EEG) obtained during the cold-pressor test. The pain scores due to the cold-pressor test decreased by 9% after the sham session, whereas the unpleasantness scores decreased by 7% after both interventions. The brain activity associated with the cold-pressor test following the sham session decreased significantly in the delta and alpha bands; and showed a marginally significant decrease in theta and beta bands, whereas there were no changes in EEG source localization following the chiropractic spinal adjustment session.

### EEG activity during tonic pain

This study found increased neural activity in all frequency bands when CP was compared with resting state. The increase in cortical activity due to tonic pain is supported by other studies using EEG^53–55^, PET^56^ and fMRI^57,58^. In this study, the brain regions with a change in activity included the insula and anterior cingulate cortex, which are among the most often reported active regions during pain perception in many neuroimaging studies using fMRI and PET^59,60^. Hence, the insula and anterior cingulate cortex likely have an important role in the processing of pain. We found increased delta, theta and beta activities, which is consistent with findings of studies utilizing tonic pain in healthy volunteers^40,53–55,61^. The increase of the delta, theta and beta activities in the anterior cingulate and insula cortices, among other regions in this study, likely implies negative feelings to the pain induced by CP, as it has been reported by many studies^58,60,62,63^ that these regions are associated with emotional aspects of pain processing.

There was an increase in alpha activity during the CP test. Alpha oscillations have been found to be increased over frontal or parieto-occipital regions during tonic pain using the CP test^53^ and hypertonic saline injection^61^. On the other hand, alpha power and underlying neural activity have also been shown to decrease following tonic pain^37,40,64^. The type or intensities of stimulus used in these studies may be the reason for inconsistency with the current study. For example, Shao *et al*. used a 10 °C CP test^40^, Babiloni *et al*. used CO2-laser stimulation^64^ and heat stimulation was used by Nir *et al*.^37^. The changes in the alpha EEG band are associated with attention processes^65^ and anticipation to pain^64^. Therefore, the increase of source activity underlying the alpha band in this study is likely due to the subjects’ attention to pain.

### Sham intervention

After the sham intervention there was a decrease in pain scores during the tonic pain. This is not an unexpected result, since habituation to pain is a normal reaction in humans and animals to continuous or repetitive painful stimuli, which decreases the perceived pain and pain-related responses^66–68^. There was also a decrease in the neural activity underlying EEG during tonic pain following the sham intervention, indicating the pain scores related to the underlying activity in these specific brain regions. The regions which showed the most significant decrease were: cingulate gyrus, limbic lobe (delta) and transverse temporal gyrus, temporal lobe (alpha). Marginally significant decrease was seen in the medial frontal gyrus, frontal lobe (theta) and superior temporal gyrus, temporal lobe (beta). The areas that showed a decrease due to the CP test are a subgroup of brain areas, which were also activated due to the CP test itself. Hence, the decrease of activity in these brain areas likely has a role in the inhibition of pain, as seen in the decreased pain scores. In this study, there were about 10 minutes between the first and second EEG recording during tonic pain. The after-effects of tonic pain can last up to 30 minutes in humans^69,70^ and therefore, the decreased cortical activity, in combination with the decreased pain/unpleasantness perception of the CP stimulus after the sham intervention, can most likely be attributed to central habituation to the CP-induced pain. It is possible that there is some form of placebo effect occurring as well, as most subjects in this study were novices to chiropractic and therefore did not know what to expect.

Although habituation to pain is a normal reaction in humans and animals to continuous or repetitive pain stimuli^66–68^, it is a multifactorial event that is not well understood^71^. It has been hypothesized that habituation actually involves dual competing processes of depression (habituation) and facilitation (sensitization), that combine to give a behavioral or perceptual outcome^72^. Habituation to pain may lead to maladaptive plastic changes in the neural system. Maladaptive neuroplasticity has been reported to be induced by chronic pain^73^. These maladaptive neuroplastic changes cause individual sufferers to experience symptoms and functional disturbance, rather than the pain itself^74–76^.

### Spinal adjustment intervention

In the present study, there were no differences in neural activity due to CP-induced pain following the spinal adjustment session. This may be due to the spinal adjustments having an impact on pain habituation. It is possible that the altered afferent input from the spine following spinal adjustments modulates the interplay between the dual competing processes of depression (habituation) and facilitation (sensitization). Previous studies that have investigated the hypoalgesic effects of spinal manipulation on temperature-induced pain have produced conflicting results^14^. Millan *et al*. suggested that this may be related to the type of pain fibers (C-fiber or A-delta fibers) that are stimulated during different testing protocols, with C-fiber mediated pain being more influenced by a spinal manipulation intervention than A-delta fiber mediated pain^14^. Previous studies have also suggested that spinal manipulation may reduce the central sensitization of pain^16,25^. This seems somewhat paradoxical based on the findings of the present study because a lessening of sensitization would be expected to result in an increase in habituation, as opposed to a decrease^72^. It is possible that instead of simply decreasing central sensitization, spinal adjustments may in some cases ‘reset’ the facilitatory and inhibitory processes associated with habituation. This may be due to spinal adjustments resulting in altered afferent paraspinal tissue input that affects the manner in which the somatosensory cortex integrates subsequent afferent information, as has been previously hypothesized^10,23,25,27^. For example, it has been shown that spinal adjustments alter processing in the prefrontal cortex^31^. The prefrontal cortex has been shown to impact the degree to which the insular cortex is activated during repeated cold pressor stimulation, thus can alter the way in which a person’s brain habituates to a cold-pain stimulus^77^. Gaining a greater understanding of these potential mechanisms and processes may be important when considering the effects of chiropractic care on both acute and chronic pain.

### Study considerations

The sham and experimental interventions were separated by up to 10 days. This may induce some time effects in the data. Furthermore, this was a pilot study with 15 subjects where the neural response to tonic pain was assessed after a single session of spinal adjustments. We recognize that the number of subjects was not high. However, due to the little variation in response and the high sensitivity of the experimental models, the numbers are within the normal range for such explorative studies^10,78–82^. To further validate these findings, future studies should look at the effects of chiropractic care on central processing of tonic pain in a larger population and over a longer period of chiropractic care.

It would also be worth considering to include a non-intervention session, as mind-set has been shown to impact habituation to cold-pain stimuli^83^. From the current study design, it is therefore hard to make firm conclusions about the sham only effects. It is impossible to be sure whether our results following the sham were due to the habituation, placebo effect, or both. Having a separate no-intervention control session would have helped elucidate this.

Finally, it would also have been interesting to analyze the gamma band for a study such as this. However, to do that, longer EEG recordings are required to facilitate the removal of the artifacts, especially those related to muscles as the spectrum of EMG overlaps that of the gamma band. The independent component analysis (ICA) can be used for this purpose but it requires approximately 20 to 30 times squared number of channels amount of data points, and it is not easy from a practical (pain) point of view to record this amount of EEG during the CP-test as most of the subjects cannot tolerate pain this long.

## Conclusion

This study showed a habituation to pain response following the sham intervention, with no changes in the neural processing of tonic pain following the chiropractic spinal adjustment session. Changes to spinal function with chiropractic spinal adjustments appears to affect the way in which the central nervous system responds to repeated pain stimuli. However, this needs to be further explored before concrete conclusions can be made. Future studies should investigate the long-term effects of chiropractic care on pain processing in sub-clinical pain populations as well as in patients suffering from acute and chronic pain.

## References

[1] Pelletier R, Higgins J, Bourbonnais D (2015). Is neuroplasticity in the central nervous system the missing link to our understanding of chronic musculoskeletal disorders?. BMC Musculoskeletal Disorders.

[2] Pud D, Granovsky Y, Yarnitsky D (2009). The methodology of experimentally induced diffuse noxious inhibitory control (DNIC)-like effect in humans. Pain.

[3] Villanueva L, Le Bars D (1995). The activation of bulbo-spinal controls by peripheral nociceptive inputs: Diffuse noxious inhibitory controls. in. Biological Research.

[4] Benarroch EE (2006). Pain-autonomic interactions. Neurological Sciences.

[5] Melzack R, Wall PD (1965). Pain mechanisms: a new theory. Science (New York, N.Y.).

[6] Fields HL, Malick A, Burstein R (1995). Dorsal horn projection targets of ON and OFF cells in the rostral ventromedial medulla. Journal of neurophysiology.

[7] Ohara PT, Vit J-P, Jasmin L (2005). Cortical modulation of pain. Cellular and Molecular Life Sciences.

[8] Heinricher MM, Tavares I, Leith JL, Lumb BM (2009). Descending control of nociception: Specificity, recruitment and plasticity. Brain Research Reviews.

[9] Goffaux P, Redmond WJ, Rainville P, Marchand S (2007). Descending analgesia - When the spine echoes what the brain expects. Pain.

[10] Haavik H, Niazi IK, Holt K, Murphy B (2017). Effects of 12 Weeks of Chiropractic Care on Central Integration of Dual Somatosensory Input in Chronic Pain Patients: A Preliminary Study. Journal of Manipulative and Physiological Therapeutics.

[11] Gay CW, Robinson ME, George SZ, Perlstein WM, Bishop MD (2014). Immediate changes after manual therapy in resting-state functional connectivity as measured by functional magnetic resonance imaging in participants with induced low back pain. Journal of Manipulative and Physiological Therapeutics.

[12] Teodorczyk-Injeyan JA, Injeyan HS, Ruegg R (2006). Spinal manipulative therapy reduces inflammatory cytokines but not substance P production in normal subjects. in. Journal of Manipulative and Physiological Therapeutics.

[13] Pickar JG (2002). Neurophysiological effects of spinal manipulation. *The spine journal official journal of the North American Spine*. Society.

[14] Millan M, Leboeuf-Yde C, Budgell B, Amorim M-A (2012). The effect of spinal manipulative therapy on experimentally induced pain: a systematic literature review. Chiropractic & manual therapies.

[15] Goertz CM, Pohlman KA, Vining RD, Brantingham JW, Long CR (2012). Patient-centered outcomes of high-velocity, low-amplitude spinal manipulation for low back pain: A systematic review. Journal of Electromyography and Kinesiology.

[16] Ruddock JK, Sallis H, Ness A, Perry RE (2016). Spinal Manipulation vs Sham Manipulation for Nonspecific Low Back Pain: A Systematic Review and Meta-Analysis. Journal of Chiropractic Medicine.

[17] Rosner AL (2016). Chiropractic Identity: A Neurological, Professional, and Political Assessment. Journal of Chiropractic Humanities.

[18] The Rubicon Group. Definition and Position Statement on the Chiropractic Subluxation. 2017 http://www.therubicongroup.org/#/policies/ 4 Available at: http://www.therubicongroup.org/#/policies/ (2017).

[19] Triano JJ (2013). Review of methods used by chiropractors to determine the site for applying manipulation. Chiropractic & manual therapies.

[20] Cooperstein, R. & Gleberzon, B. J. *Technique Systems in Chiropractic*. (Churchill Livingstone 2004).

[21] Holt K, Kelly B, Taylor HH (2009). Practice Characteristics of Chiropractors in New Zealand. Chiropractic Journal of Australia.

[22] Mintken PE, Derosa C, Little T, Smith B (2008). & American Academy of Orthopaedic Manual Physical Therapists. A model for standardizing manipulation terminology in physical therapy practice. The Journal of manual & manipulative therapy.

[23] Haavik H, Murphy B (2012). The role of spinal manipulation in addressing disordered sensorimotor integration and altered motor control. Journal of Electromyography and Kinesiology.

[24] Bialosky JE, Bishop MD, Price DD, Robinson ME, George SZ (2009). The mechanisms of manual therapy in the treatment of musculoskeletal pain: A comprehensive model. Manual Therapy.

[25] Bishop MD, Beneciuk JM, George SZ (2011). Immediate reduction in temporal sensory summation after thoracic spinal manipulation. Spine Journal.

[26] Haavik Taylor H, Murphy BA (2007). Altered cortical integration of dual somatosensory input following the cessation of a 20 min period of repetitive muscle activity. Experimental Brain Research.

[27] Haavik Taylor H, Holt K, Murphy B (2010). Exploring the neuromodulatory effects of the vertebral subluxation and chiropractic care. Chiropractic Journal of Australia.

[28] Murphy B, Taylor HH, Marshall P (2010). The Effect of Spinal Manipulation on the Efficacy of a Rehabilitation Protocol for Patients With Chronic Neck Pain: A Pilot Study. Journal of Manipulative and Physiological Therapeutics.

[29] Baarbé J (2014). A novel protocol to investigate motor training-induced plasticity and sensorimotor integration in the cerebellum and motor cortex. Journal of neurophysiology.

[30] Niazi IK (2015). Changes in H-reflex and V-waves following spinal manipulation. Experimental Brain Research.

[31] Lelic D (2016). Manipulation of Dysfunctional Spinal Joints Affects Sensorimotor Integration in the Prefrontal Cortex: A Brain Source Localization Study. Neural plasticity.

[32] Srbely J (2010). Chiropractic science: a contemporary neurophysiologic paradigm. The Journal of the Canadian Chiropractic Association.

[33] Learman KE (2009). Effects of Spinal Manipulation on Trunk Proprioception in Subjects With Chronic Low Back Pain During Symptom Remission. Journal of Manipulative and Physiological Therapeutics.

[34] Haavik H, Murphy B (2011). Subclinical neck pain and the effects of cervical manipulation on elbow joint position sense. Journal of Manipulative and Physiological Therapeutics.

[35] Huber MT, Bartling J, Pachur D, Woikowsky-Biedau Sv, Lautenbacher S (2006). EEG responses to tonic heat pain. Experimental brain research.

[36] Nir R-R, Sinai A, Raz E, Sprecher E, Yarnitsky D (2010). Pain assessment by continuous EEG: association between subjective perception of tonic pain and peak frequency of alpha oscillations during stimulation and at rest. Brain research.

[37] Nir R-R, Sinai A, Moont R, Harari E, Yarnitsky D (2012). Tonic pain and continuous EEG: prediction of subjective pain perception by alpha-1 power during stimulation and at rest. Clinical neurophysiology: official journal of the International Federation of Clinical Neurophysiology.

[38] Gram M, Graversen C, Olesen SS, Drewes AM (2015). Dynamic spectral indices of the electroencephalogram provide new insights into tonic pain. Clinical Neurophysiology.

[39] Rainville P, Feine JS, Bushnell MC, Duncan GH (1992). A psychophysical comparison of sensory and affective responses to four modalities of experimental pain. Somatosensory & motor research.

[40] Shao S, Shen K, Yu K, Wilder-Smith EPV, Li X (2012). Frequency-domain EEG source analysis for acute tonic cold pain perception. Clinical Neurophysiology.

[41] Jones AKP, Kulkarni B, Derbyshire SWG (2003). Pain mechanisms and their disorders. British Medical Bulletin.

[42] Apkarian AV, Bushnell MC, Treede RD, Zubieta JK (2005). Human brain mechanisms of pain perception and regulation in health and disease. European Journal of Pain.

[43] Leone M (2006). Neuroimaging and pain: A window on the autonomic nervous system. Neurological Sciences.

[44] Pascual-Marqui RD (2002). Standardized low-resolution brain electromagnetic tomography (sLORETA): technical details. Methods and findings in experimental and clinical pharmacology.

[45] Khodayari-Rostamabad A (2015). A cortical source localization analysis of resting EEG data after remifentanil infusion. Clinical Neurophysiology.

[46] Hansen TM (2017). Characterization of cortical source generators based on electroencephalography during tonic pain. Journal of Pain Research.

[47] Lelic D, Hansen TM, Mark EB, Olesen AE, Drewes AM (2017). The effects of analgesics on central processing of tonic pain: A cross-over placebo controlled study. Neuropharmacology.

[48] Holt KR (2009). Interexaminer Reliability of a Leg Length Analysis Procedure Among Novice and Experienced Practitioners. Journal of Manipulative and Physiological Therapeutics.

[49] Mazziotta J (2001). A probabilistic atlas and reference system for the human brain: International Consortium for Brain Mapping (ICBM). Philosophical transactions of the Royal Society of London. Series B, Biological sciences.

[50] Oostenveld, R., Fries, P., Maris, E. & Schoffelen, J. M. FieldTrip: Open source software for advanced analysis of MEG, EEG, and invasive electrophysiological data. *Computational Intelligence and Neuroscience* **2011** (2011). 10.1155/2011/156869 PMC3021840 21253357

[51] Nichols TE, Holmes AP (2002). Nonparametric permutation tests for functional neuroimaging: A primer with examples. Human Brain Mapping.

[52] Maris E, Oostenveld R (2007). Nonparametric statistical testing of EEG- and MEG-data. Journal of Neuroscience Methods.

[53] Backonja M (1991). Tonic changes in alpha power during immersion of the hand in cold water. Electroencephalography and Clinical Neurophysiology.

[54] Ferracuti S, Seri S, Mattia D, Cruccu G (1994). Quantitative EEG modifications during the Cold Water Pressor Test: hemispheric and hand differences. International journal of psychophysiology: official journal of the International Organization of Psychophysiology.

[55] Chang PF, Arendt-Nielsen L, Chen ACN (2002). Dynamic changes and spatial correlation of EEG activities during cold pressor test in man. Brain Res Bull.

[56] Casey KL (1999). Forebrain mechanisms of nociception and pain: analysis through imaging. Proceedings of the National Academy of Sciences of the United States of America.

[57] Frankenstein UN, Richter W, McIntyre MC, Rémy F (2001). Distraction modulates anterior cingulate gyrus activations during the cold pressor test. NeuroImage.

[58] Wager TD (2013). An fMRI-based neurologic signature of physical pain. The New England journal of medicine.

[59] Peyron, R., Laurent, B. & García-Larrea, L. Functional imaging of brain responses to pain. A review and meta-analysis (2000). *Neurophysiologie Clinique/Clinical Neurophysiology* **30**, 263–288 (2000). 10.1016/s0987-7053(00)00227-6 11126640

[60] Zhuo M (2008). Cortical excitation and chronic pain. Trends in Neurosciences.

[61] Le Pera D (2000). Long-lasting effect evoked by tonic muscle pain on parietal EEG activity in humans. Clinical Neurophysiology.

[62] Craig A, Reiman E, Evans A, Bushnell M (1996). Functional imaging of an illusion of pain. Nature.

[63] Tracey I, Mantyh PW (2007). The Cerebral Signature for Pain Perception and Its Modulation. Neuron.

[64] Babiloni C (2006). Anticipatory Electroencephalography Alpha Rhythm Predicts Subjective Perception of Pain Intensity. Journal of Pain.

[65] Klimesch W (1999). *EEG alpha and theta oscillations reflect cognitive and memory performance: A review and analysis*. Brain Research Reviews.

[66] LeBlanc J, Potvin P (1966). Studies on habituation to cold pain. Canadian journal of physiology and pharmacology.

[67] Strempel H (1976). Adaptive modifications of cold pain (author’s transl). European journal of applied physiology and occupational physiology.

[68] Strempel H (1978). Adaptive modifications of cold pain. III. Communication: short-term experiments with 1-min-intervals (author’s transl). European journal of applied physiology and occupational physiology.

[69] Farina S (2001). Transient inhibition of the human motor cortex by capsaicin-induced pain. A study with transcranial magnetic stimulation. Neuroscience Letters.

[70] Valeriani M (2005). Inhibitory effect of capsaicin evoked trigeminal pain on warmth sensation and warmth evoked potentials. Experimental Brain Research.

[71] Coppola G, Di Lorenzo C, Schoenen J, Pierelli F (2013). Habituation and sensitization in primary headaches. The journal of headache and pain.

[72] Thompson RF (2009). Habituation: A history. Neurobiology of Learning and Memory.

[73] Seifert F, Maihöfner C (2011). Functional and structural imaging of pain-induced neuroplasticity. Current Opinion in Anaesthesiology.

[74] Brumagne S, Cordo P, Lysens R, Verschueren S, Swinnen S (2000). The role of paraspinal muscle spindles in lumbosacral position sense in individuals with and without low back pain. Spine.

[75] Michaelson P (2003). Vertical posture and head stability in patients with chronic neck pain. Journal of Rehabilitation Medicine.

[76] Paulus I, Brumagne S (2008). Altered interpretation of neck proprioceptive signals in persons with subclinical recurrent neck pain. Journal of Rehabilitation Medicine.

[77] Bogdanov VB (2015). Cerebral responses and role of the prefrontal cortex in conditioned pain modulation: An fMRI study in healthy subjects. Behavioural Brain Research.

[78] Jochumsen M, Niazi IK, Nedergaard RW, Navid MS, Dremstrup K (2018). Effect of subject training on a movement-related cortical potential-based brain-computer interface. Biomedical Signal Processing and Control.

[79] Lelic D, Olesen SS, Valeriani M, Drewes AM (2012). Brain source connectivity reveals the visceral pain network. NeuroImage.

[80] Jochumsen M (2015). Detecting and classifying movement-related cortical potentials associated with hand movements in healthy subjects and stroke patients from single-electrode, single-trial EEG. Journal of neural engineering.

[81] Jochumsen M (2015). Online multi-class brain-computer interface for detection and classification of lower limb movement intentions and kinetics for stroke rehabilitation. Brain-Computer Interfaces.

[82] Jochumsen M (2018). Investigation of Optimal Afferent Feedback Modality for Inducing Neural Plasticity with A Self-Paced Brain-Computer Interface. Sensors (Basel, Switzerland).

[83] Smith BW (2009). The Role of Resilience and Purpose in Life in Habituation to Heat and Cold Pain. Journal of Pain.

